# Dentoalveolar Procedures in Immune Thrombocytopenia; Systematic Review and an Institutional Guideline

**DOI:** 10.1055/a-1641-7770

**Published:** 2021-09-09

**Authors:** Wobke E. M. van Dijk, Robert J. J. van Es, Maria E. P. Correa, Roger E. G. Schutgens, Karin P. M. van Galen

**Affiliations:** 1 Center for Benign Hematology, Thrombosis and Hemostasis, Van Creveldkliniek, University Medical Center Utrecht, Utrecht, The Netherlands; 2 Department of Oral and Maxillofacial Surgery, University Medical Center Utrecht, Utrecht, The Netherlands; 3 Oral Medicine Ambulatory, Hematology and Hemotherapy Center, University of Campinas, Campinas, Sao Paulo, Brazil

**Keywords:** dentoalveolar, ITP, immune thrombocytopenia, dental surgery, oral care

## Abstract

**Background** Dentoalveolar procedures in immune thrombocytopenia (ITP) pose a risk of bleeding due to thrombocytopenia and infection due to immunosuppressive treatments. We aimed to systematically review the safety and management of dentoalveolar procedures in ITP patients to create practical recommendations.

**Methods** PubMed, Embase, Cochrane, and Cinahl were searched for original studies on dentoalveolar procedures in primary ITP patients. We recorded bleeding- and infection-related outcomes and therapeutic strategies. Clinically relevant bleeding was defined as needing medical attention.

**Results** Seventeen articles were included, of which 12 case reports/series. Overall, the quality of the available evidence was poor. Outcomes and administered therapies (including hemostatic therapies and prophylactic antibiotics) were not systematically reported. At least 73 dentoalveolar procedures in 49 ITP patients were described. The range of the preoperative platelet count was 2 to 412 × 10 ^9^ /L. Two clinically relevant bleedings (2%) were reported in the same patient of which one was life-threatening. Strategies used to minimize the risk of bleeding were heterogeneous and included therapies to increase platelet count, antifibrinolytics, local measures, and minimally invasive techniques. Reports on the occurrence of bleedings due to anesthetics or infection were lacking.

**Conclusion** Based on alarmingly limited data, clinically relevant bleedings and infections after dentoalveolar procedures in ITP patients seem rare. Awaiting prospective and controlled studies to further evaluate these risks and the efficacy of therapeutic interventions, we provided our institutional guideline to guide the management of dentoalveolar procedures in ITP patients.

## Introduction

Oral health care is an important part of general health. Dentoalveolar procedures, which include any surgical or nonsurgical oral or dental procedure, pose a risk of bleeding. 1 Platelets play a crucial role in maintaining hemostasis in the alveolar crest and the well-vascularized oral mucosa. 2 3 In immune thrombocytopenia (ITP), the risk of bleeding is therefore increased. 4 ITP is a disorder in which autoantibodies destruct platelets and impair platelet production, leading to persistent thrombocytopenia (<100 × 10 ^9^ /L platelets). 5 6 Treatments for ITP focus on inhibiting the immune response or increasing platelet production. Complete (spontaneous) remission is possible 6 but (severe) thrombocytopenia remains a problem during the acute phase, relapses, and in refractory patients. 7 Furthermore, the platelet function might also be affected, resulting sometimes in an unpredictable bleeding tendency. 8 In thrombocytopenic patients (due to any etiology), the risk of postoperative bleeding after dentoalveolar procedures is approximately 4.9%, 9 five times higher than the 0.2 to 1.4% in healthy individuals. 10 11

Postoperative bleeding after dentoalveolar procedures can vary from being inconvenient if there is a need for reassessment, discomfort, or infection of the hematoma of being life-threatening and if the bleeding involves the floor of the mouth and thereby obstructs the upper airway. Few types of procedures pose a significant bleeding risk, but risks vary largely as per procedure. 12 13 For example, single extractions and limited endodontic surgery are generally accepted as low-risk, while multiple extractions, especially upper molars, and extensive invasive osseous surgery are considered high risk. 1 12 13 For many procedures, the exact bleeding risk is unknown, and also depends on other factors such as the presence of periodontal disease, age, and comorbidity of the patient. 9 14 15 16 17 18

There are no guidelines to support dental professionals and hematologists in the management of dentoalveolar procedures in ITP patients. Methods to prevent postoperative bleeding in thrombocytopenic patients include local hemostatic techniques (primary closure, minimally traumatizing techniques, the use of hemostatic sponges, and fibrin sealants), antifibrinolytics, and increasing the platelet count. As minimal platelet count for invasive dentoalveolar procedures in thrombocytopenic patients of any etiology, a count of >50 × 10 ^9^ /L has previously been recommended to avoid bleeding, 1 13 14 19 20 although low-risk procedures might be safely performed at >30 × 10 ^9^ /L and routine noninvasive dentistry at >10 × 10 ^9^ /L. 12 21 22 However, these recommendations are not validated, do not distinguish specific dentoalveolar procedures, are generally not specific for ITP, and are based on expert opinion or consensus. In addition, no evidence-based guidelines are available for the use of antifibrinolytics and local hemostatic techniques in this patient category.

Of note, ITP patients might be at risk of infections in addition to bleeding. First, because ITP treatment is often based on immunosuppression. Particularly corticosteroids are known to inhibit the immune response, as well as wound healing, which is essential to prevent infectious problems. 23 24 Furthermore, ITP patients are prone to having poor oral hygiene due to bleeding complaints related to tooth brushing. 25 26 Lastly, platelets play a (not fully explored) role in immune responses. 27 The exact risk of infection is unknown. No guidelines advise on the use of antibiotic prophylaxis for dentoalveolar procedures in ITP patients.

We systematically reviewed the available literature on the risk of dentoalveolar procedures in primary ITP, with respect to bleeding and infection and the effectiveness of management strategies. Since dentoalveolar procedures in ITP patients are a daily practice, we aimed to provide guidelines to guide dental professionals and hematologists in the management.

## Methods

The databases, that is, PubMed, Embase, Cochrane, and Cinahl, were searched up to April 23, 2020. The search strategy combined all key terms for ITP, dental/oral/tooth and procedures/surgery (see Search Details in Supplementary Material for the detailed search). The references of relevant articles were searched for any additional papers. All original studies in English or Dutch assessing dentoalveolar procedures in adult and pediatric patients with known ITP at the time of procedure were included if the full text was available. We excluded papers about ITP secondary to an underlying disease, as well as papers about major maxillofacial surgery or exclusively orthodontic procedures. Data were extracted using a standardized form and included information about the ITP specifics; procedure; anesthetic methods; pre-, peri-, and postoperative preventive strategies; and bleeding- and infection-related outcomes. The included articles were critically appraised on the quality of the available data and, if applicable, on the risk of bias, using the Cochrane Robins-I tool.

To include all potentially relevant evidence, we included any article where the patient(s) was/were regarded to have ITP, independent of how the diagnosis was made. Furthermore, data for adults and children were reported separately; if data on age were missing, the data are reported in the adults section.

Dentoalveolar procedures were defined as any surgical or nonsurgical oral or dental procedure except major maxillofacial surgery or exclusively orthodontic procedures. The search included a variety of both surgical and nonsurgical dentoalveolar procedures with a potential bleeding risk, such as tooth extractions, submucosal scaling, and periodontal, endodontic, restorative, and orthodontic treatment. An explanation of specific dental procedures and terminology is provided in Supplementary Table S1 .

Any reported bleeding episode was recorded and categorized according to the American College of Chest Physicians guidelines. 28 A bleeding was considered not clinically relevant if the bleeding was self-limiting (e.g., with local pressure) and did not require medical attention. Clinically relevant bleedings were divided into major if the bleeding required transfusion (of ≥2 units) of red blood cells, and nonmajor if the bleeding was not major but required medical attention (e.g., application of wound dressing or additional sutures). We assumed that no clinically relevant bleeding occurred if it was not reported in the paper. If possible, data were recorded separately for each individual dentoalveolar procedure but in some papers, only summarized data was available.

Any given therapies to decrease the risk of bleeding were recorded. These included antifibrinolytic therapies and local/surgical measures (e.g., fibrin sealants and splints), as well as therapies aiming to increase the platelet count preprocedurally, and any ITP medication that patients were on independent of the dentoalveolar procedure.

We provided the institutional guideline as used in the Van Creveldkliniek, expert center on benign hematology and hemostasis of the University Medical Center Utrecht, the Netherlands. This guideline is based on the available evidence and, where the evidence proved to be limited, the clinical experience from the Van Creveldkliniek.

## Results

**Fig. 1:**
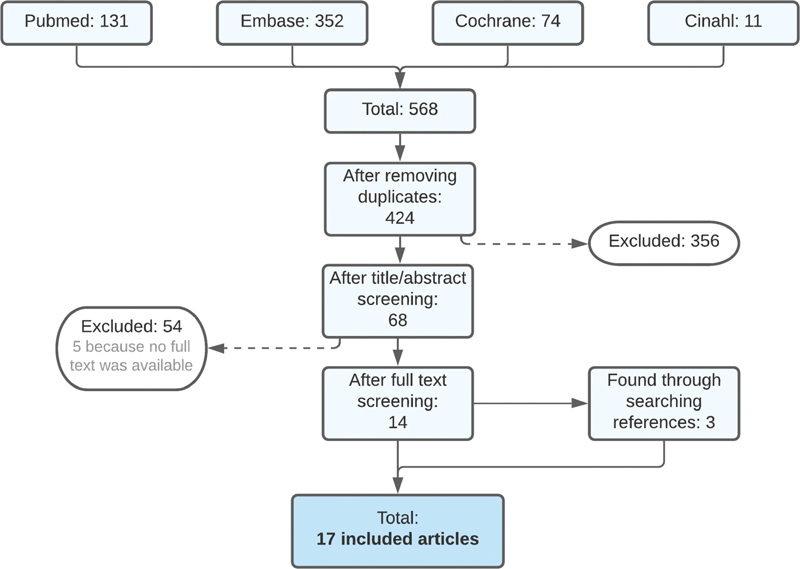
Flowchart article screening.

The search yielded 424 unique articles of which 14 articles were selected ( Fig. 1 ). Additionally, by searching references, three extra studies were found that all assessed patients with a variety of bleeding disorders of whom a few had ITP. Five articles were excluded because the full text was unavailable. In total, 17 articles were included in our review. The details of the selected studies are reported in Table 1 . Twelve articles were case reports/series, four were prospective one-arm studies that included a subset of ITP patients, and one was a retrospective cohort study. Only one article clearly reported criteria for the ITP diagnosis.

**Table 1:** Included articles

Study (year)	Study design	Number of cases	Criteria for ITP diagnosis	Number of procedures	Completeness of case description	Quality of the study (risk of bias) c	Potential conflicts of interest
Baudo et al (1985) 29	Prospective trial, one arm a	4 a (NR)	NR	6 b	Moderate	Medium	NR
Bartholomew et al (1989) 32	Prospective trial, one arm a	1 a (adult)	NR	1	Moderate	High	NR
Finucane et al (2004) 89	Case report	1 (child)	NR	2	Good	NA	NR
Guzeldemir (2009) 90	Case report	1 (child)	After BM biopsy	3	Good	NA	None
Harms et al (1996) 91	Case report	1 (adult)	NR	1	Moderate	NA	NR
Inchingolo et al (2005) 92	Case series	1 (adult)	NR	1	Moderate	NA	NR
Larionova et al (2019) 93	Case report	1 (adult)	NR	1	Good	NA	None
Lee et al (2018) 33	Case report	1 (adult)	NR	3	Good	NA	None
Martin et al (2013) 94	Case series	1 (adult)	NR	1	Good	NA	NR
McKelvy et al (1976) 34	Case report	1 (adult)	After BM biopsy	1	Good	NA	NR
Oda, 2002 95	Case report	1 (child)	NR	3	Good	NA	NR
Rakocz et al (1993) 30	Prospective trial, one arm a	6 a (NR)	NR	11 b	Moderate	Medium	NR
Sangwan et al (2013) 96	Case report	1 (adult)	NR	1	Good	NA	NR
Suwannuraks et al (1999) 31	Prospective trial, one arm a	1 a (NR)	NR	2 b	Moderate	Medium	NR
Tarantino et al (2015) 55	Retrospective cohort	24 (adults)	According to ASH/BCSH guidelines	32	Inadequate	High	Potential d
Tay et al (2013) 97	Case report	1 (child)	NR	1	Good	NA	NR
Vaisman et al (2004) 98	Case series	2 (children)	NR	3	Moderate	NA	NR

Abbreviations: ASH, American Society for Hematology ; BCSH, British Committee for Standards in Hematology ; BM, bone marrow ; ITP, immune thrombocytopenia; NA, not applicable ; NR, not reported.

a Subset of ITP patients in cohort of patients with bleeding disorders.

b Number of extracted teeth rather than procedures.

c Not applicable for case reports.

d The work was supported by GlaxoSmithKline (GSK). Tarantino provided consultancy for Amgen, Fogarty received funding from GSK and provided consultancy for Amgen, Shah is an employee of and holds equity ownership in GSK, and Brainsky is a former employee of and held equity ownership in GSK.

Fig. 1 shows article screening procedure through a flowchart.

In this review, a total of 73 dentoalveolar procedures were performed in 49 ITP patients of which 13 of 73 (18%) procedures in 6 of 49 (12%) children ( Table 1 ). Critical appraisal revealed that the quality of the overall available evidence was poor ( Supplementary Tables S2 , S3 and S4 ), partly because the majority of articles comprised case reports. The four prospective studies and one retrospective cohort study had a medium-to-high risk of bias. Of the prospective studies, three assessed the effectiveness of local hemostatic measures 29 30 31 and one of aminocaproic acid. 32 None of the studies had a control group.

### Bleeding Complications

Overall, two clinically relevant bleedings, - one major and one nonmajor, were reported after 2 of 60 (3%) procedures in adults, both in the same patient (1/43 [2%] of the adults). 33 None of the children experienced a clinically relevant bleeding. The first bleeding, nonmajor, occurred after extraction of the left mandibular first molar, at a platelet count of 35 × 10 ^9^ /L. It persisted for 1 week and was controlled by extra suturing. The second bleeding, major, occurred after dental implantation of that same molar. It happened at a platelet count of 22 × 10 ^9^ /L and presented with submandibular swelling within 24 hours after the procedure. The bleeding was complicated by an airway obstruction for which the patient received an emergency tracheostomy and a decrease in hemoglobin of 3 g/dL for which the patient received red blood cell transfusions. No therapies were administered to increase the platelet count. The authors failed to mention whether preventive hemostatic measures were applied.

Tables 2 and 3 summarize which procedures were reported in the selected articles at which platelet count for adults and children separately and whether a bleeding occurred. The largest proportion of the procedures comprised dental extractions (29/73 [40%]), mostly regular dental extractions in adults (25/29 [86%]). In 5 of 29 (17%), the extraction was performed in a child and combined with another procedure. No bleeding occurred in the 14 of 73 (19%) procedures where the platelet count was <20 × 10 ^9^ /L. Procedures with a platelet count <20 × 10 ^9^ /L were performed mainly in adults; 12 procedures were specifically minimally invasive. The only procedure performed in a child at this low platelet count was because of the patient was refractory to all available treatments. Of note, for 38 of 73 (52%) procedures, only summarized data were available on the platelet count before the procedure and the outcomes.

**Table 2:** Type of dentoalveolar procedure, platelet count at time of procedure, and therapies to increase platelet count preoperatively: adults

		Platelet count at time of procedure							Total per type of procedure (row total)	Number of clinically relevant bleedings *n* (% of row total)	
		Categories				Reported otherwise		Not reported		
		<20 × 10 ^9^ /L	20–50 × 10 ^9^ /L	50–100 × 10 ^9^ /L	>150 × 10 ^9^ /L	10–70 a × 10 ^9^ /L	80 b × 10 ^9^ /L (9–412) a			
All procedures *n* (%)		13 (22)	2 c d (3)	4 (7)	1 (2)	6 (10)	32 (53)	2 (3)	60	2 (3)
Type of procedure, *n* (% of column total)										
Cleaning procedures				1 (25)					1	0 (0)
	Scaling, root planing, debridement			1 e					1	0 (0)
Combined procedures		1 (8)							1	0 (0)
	Reendodontic treatment + incision of subperiosteal abscess using laser	1 e							1	0 (0)
Dental extraction(s)		12 (92)	1 c (50)	3 (75)	1 (100)	6 (100)		1 (50)	24	1 (4)
	Regular		1 c	3 e ^in 2^	1	6			11	1 (9)
	Minimally invasive	12						1	13	0 (0)
Dental implantation			1 d (50)					1 (50)	2	1 (50)
	Regular		1 d						1	1 (100)
	Prosthetic implant restoration							1	1	0 (0)
Other							32 (100)		32	0 (0)
	Several procedures including cleaning, crowns, prosthetics, dental extraction, and endodontics						32 e ^in 5^		32	0 (0)
Strategies used preoperatively to raise platelet count, *n* (% of column total)										
Any		1 (8)		4 (100)			5 (16)		10	0 (0)
	Prednisone/methylprednisolone	1		2			1		4	0 (0)
	Hydrocortisone + platelet transfusion						1		1	0 (0)
	IVIg			1			2		3	0 (0)
	Eltrombopag			1					1	0 (0)
	Danazol + platelet transfusion						1		1	0 (0)
None		11 (85)	2 c d (100)			6 (100)	27 (84)		46	2 (4)
NR		1 (8)			1 (100)			2 (100)	4	0 (0)

Abbreviation: IVIg, intravenous immunoglobulin.

a Range.

b Median.

c Clinically relevant nonmajor bleeding occurred.

d Major bleeding occurred.

e Indicates that therapies were given in advance to increase the platelet count. The reported platelet counts at the time of procedure were after any therapies were given.

**Table 3:** Type of dentoalveolar procedure, platelet count at time of procedure, and therapies to increase platelet count preoperatively: children

		Platelet count at time of procedure						Total per type of procedure (row total)	Number of clinically relevant bleedings *n* (% of row total)
		Categories					Not reported		
		<20 × 10 ^9^ /L	20–50 × 10 ^9^ /L	50–100 × 10 ^9^ /L	100–150 × 10 ^9^ /L	>150 × 10 ^9^ /L			
All procedures *n* (% of row total)		1 (8)	2 (15)	1 (8)	2 (15)	1 (8)	6 (46)	13	0 (0)
Type of procedure, *n* (% of column total)									
Cleaning procedures					1 (50)	1 (50)	1 (17)	3	0 (0)
	Scaling, root planing, debridement				1	1		2	0 (0)
	Cleaning in severely neglected oral health status						1	1	0 (0)
Combined procedures		1 (100)	1 (50)				3 (50)	5	0 (0)
	Dental extraction(s) + cleaning and scaling	1						1	0 (0)
	Dental extraction(s) + composite reconstruction + amalgam restoration + pulpotomy and crown						1	1	0 (0)
	Dental extraction(s) + incisional biopsy						1 ^c^	1	0 (0)
	Dental extraction(s) + mandibular fenestration of simple bone cyst		1 a				1 ^c^	2	0 (0)
Trauma procedures				1 (100)				1	0 (0)
	Repositioning and splinting of traumatically displaced alveolus and teeth			1 ^c^				1	0 (0)
Endodontic procedures			1 (50)				1 (17)	2	0 (0)
	Endodontic treatment of nonvital replaced teeth						1	1	0 (0)
	Composite reconstruction + pulpotomy		1					1	0 (0)
Restorative and orthodontic procedures					1 (50)		1 (17)	2	0 (0)
	Final rehabilitation (crowns, prosthetic space maintainers)				1 a			1	0 (0)
	Pit and fissure sealing + composite restorations + orthodontics						1	1	0 (0)
Strategies used preoperatively to raise platelet count, *n* (% of column total)									
Any			1 (50)		1 (50)		2 (33)	4	0 (0)
	Prednisone/methylprednisolone				1			1	0 (0)
	IVIg + platelet transfusion		1				1	2	0 (0)
	Platelet transfusion						1	1	0 (0)
None		1 (100)		1 (100)				2	0 (0)
Not reported			1 (50)		1 (50)	1 (100)	4 (67)	7	0 (0)

Abbreviation: IVIg, intravenous immunoglobulin.

a Indicates that therapies were given in advance to increase the platelet count. The reported platelet counts at the time of procedure were after any therapies were given.

Therapies aimed to increase the platelet count were given prior to 10 of 60 (17%) procedures in adults and 4 of 13 (31%) in children ( Tables 2 and 3 ). The initial platelet counts in these patients ranged from 2 to 48 × 10 ^9^ /L. The most common strategies were steroids and intravenous immunoglobulins (IVIg), with or without platelet transfusion. Eltrombopag was only given once, in an adult, which increased the platelet count from 18 to 55 × 10 ^9^ /L. Romiplostim was not used. Doses and treatment durations (if reported) can be found per individual procedure in Supplementary Table S7 . Despite therapy, the platelet count was still <50 × 10 ^9^ /L at the time of two procedures but no bleeding occurred. These procedures were reendodontic treatment combined with incision of a subperiosteal abscess using laser treatment (platelet count 2 × 10 ^9^ /L after treatment with intravenous prednisone) and dental extraction combined with mandibular fenestration (platelet count 43 × 10 ^9^ /L after treatment with IVIg and platelet transfusion).

In most procedures (46/60 [77%]) in adults, 2/13 [15%] in children), no therapy was given to increase the platelet count (not reported in 7 and 54%, respectively). In the group with a platelet count of <20 × 10 ^9^ /L, no therapy was given prior to the 12 minimally invasive dental extractions after which no bleeding occurred. In the adult group with a platelet count between 20 and 50 × 10 ^9^ /L, no therapy was given prior to 2 of 2 procedures in the same patient after which the above mentioned clinically relevant bleeding events occurred.

With respect to hemostatic strategies, the use of antifibrinolytics or surgical hemostatic measures was only reported in 22 of 60 (37%) of the procedures in adults and 1 of 13 (8%) of the procedures in children. The combination of strategies was heterogeneous, as well as the route of administration and the dosing of the antifibrinolytics, if reported at all. The specific drug was tranexamic acid in all except one procedure and was administered locally (mouthwash), orally, and/or intravenously. The details per procedure can be found in Supplementary Table S7 . None of the patients in whom any hemostatic strategy was given experienced a clinically relevant bleeding ( Supplementary Tables S5–S7 ).

No bleeding complications due to the anesthetics were mentioned in the articles. In 17 of 60 (28%) of the procedures in adults and 10 of 13 (70%) of the procedures in children, the authors mentioned the anesthetic approach, that is, local infiltration anesthesia in most (14/17 [82%] in adults and 5/10 [50%] in children). Regional mandibular block anesthesia was used only in one procedure in an adult, with a platelet count between 50 and 100 × 10 ^9^ /L. The platelet count in relation to the anesthetic approach can be found in Supplementary Tables S5 and S6 .

### Infectious Complications

Neither the occurrence nor the absence of infections was mentioned in the included studies. Only in one case with a fluctuating dose of prednisolone, a “febrile episode” was reported without any other complications. 34 None of the articles reported whether antibiotic prophylaxis was administered. The use of immunosuppressive agents during the procedure was rare. In 11 of 73 (15%) procedures, patients were known to use immunosuppressive agents. All these used steroids were either administered preoperatively to perform the procedure (six procedures; Table 2 ) or as low-dose concomitant ITP medication (five procedures). Prednisolone was combined with cyclosporin A in three procedures. No other use of immunosuppressive agents was reported.

## Discussion

This systematic review showed that little evidence on the management of dentoalveolar procedures in ITP patients is available, especially in children. The limited evidence suggests that clinically relevant bleeding complications after dentoalveolar and anesthetic procedures are rare (3% of the procedures in adults and 0% of the procedures in children). However, no conclusions can be drawn concerning the effectiveness of different bleeding preventive strategies, nor the risk of infection.

This is the first systematic review regarding the management of dentoalveolar procedures in ITP patients. Strengths of the review are the structured and detailed overview of data, as well as the extensive search. Predefined inclusion and exclusion criteria and a standardized data-extraction form were used to minimize bias. An important limitation of the review is the low quality of the evidence and the few cases with truly low platelet counts (below the 30–50 × 10 ^9^ /L). Furthermore, selective reporting of outcomes, as well as the absence of standardized definitions, imposes a significant risk of bias. Finally, most articles did not explain how the ITP was diagnosed which may have caused heterogeneity of the population and limited generalizability; particularly in case of accidental inclusion of secondary ITP patients. However, the heterogeneous population may be a better reflection of the daily clinical practice where the medical history is often limited to “has ITP.”

Only two clinically relevant bleeding complications (2%) were reported, one major and one nonmajor, both in the same patient. This suggests that the risk in ITP patients is lower than the 4.9% in thrombocytopenic patients of any etiology. 9 The bleedings occurred after low bleeding risk procedures with a platelet count of 22 and 35 × 10 ^9^ /L, respectively. Based on the available ITP guidelines, these platelet counts should have been relatively safe, >10 10 ^9^ /L for routine noninvasive dentistry, >30 × 10 ^9^ /L for tooth extractions and regional mandibular blocks, and >50 × 10 ^9^ /L for minor oral surgery. 21 However, the bleeding risk is not only dependent on the platelet count 9 but also on local hemostatic interventions, the administration of antifibrinolytic therapy, and the presence of personal risk factors such as the presence of predominantly mucosal rather than cutaneous bleeding, concomitant bleeding disorders or platelet abnormalities, and comorbidity. 9 15 16 17 18 35 36 37 38 39 40 Unfortunately, in the studies selected for our review, information on bleeding manifestations and comorbidity was too limited to address this issue, and information on platelet abnormalities was absent altogether. Also in this particular patient, these factors were unknown but they could have played a significant role in the occurrence of the bleedings.

The risk of infection-related complications seems low, considering that none of the reviewed 118 procedures reported any. Of note, infections were not of primary interest in the included papers nor was the administration of antibiotic prophylaxis reported in any of the cases. ITP patients have several reasons for an increased risk of infection, including the use of immunosuppressants 23 24 41 42 43 and possible issues with oral hygiene due to bleeding complaints. 25 26 Prospective research is needed to evaluate the exact risk of dental infections in ITP patients and the need for antibiotic prophylaxis.

None of the reports mentioned bisphosphonate-related osteonecrosis of the jaw (BRONJ). BRONJ is a rare but serious condition triggered by invasive dentoalveolar procedures 44 and characterized by exposed jaw bone surrounded by inflamed tissue that can lead to osteolysis, pathological fractures, and fistulas. 45 Since bisphosphonates are sometimes administrated to ITP patients in case of long-term corticosteroids treatments, awareness is due.

### Unanswered Questions and Future Research

First and foremost, prospective studies are needed to evaluate the safety of dentoalveolar procedures in ITP patients with low platelet counts, as well as the need for and efficacy of therapeutic interventions to prevent bleeding and infection. Furthermore, to improve the individual risk assessment, other markers that predict bleeding could be of value, rather than or in addition to platelet count.

## Institutional Guideline

Dentoalveolar procedures are part of daily clinical practice. There is limited evidence on how to proceed in patients with ITP. Therefore, we provide our institutional guideline which combines the preceding literature review with our clinical experience. The guideline is primarily intended for adults, particularly concerning the recommendations about raising the platelet count, and are used in our expertise center for benign hematology and hemostasis (Van Creveldkliniek, University Medical Center Utrecht, The Netherlands).

### Bleeding

**Fig. 2:**
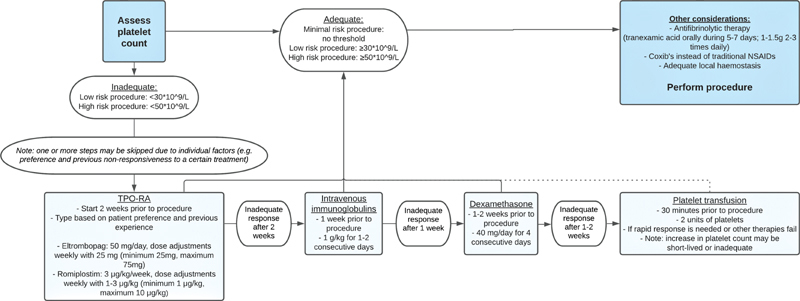
Flowchart to prepare dentoalveolar procedure. NSAIDs, nonsteroid anti-inflammatory drugs, TPO-RA, thrombopoietin-receptor agonists.

Measures to prevent bleeding may include appropriate planning of the procedure, pharmacological treatment to increase the platelet count and improve hemostasis, as well as and nonpharmacological interventions to achieve local hemostasis. An overview of the recommendations is depicted in the flowchart ( Fig. 2 ). Different aspects of this flowchart are discussed in more detail below.

To improve future research and management, a uniform classification of postoperative bleeding after dentoalveolar procedures is mandatory. As an ITP-specific classification is unavailable, we recommend the internationally accepted and widely used classification from the American College of Chest Physicians guidelines. This classification divides bleedings into “major,” “clinically relevant nonmajor,” and “minor.” 28 The exact definition is provided in the “Methods” section of this article.

### Bleeding Risk and Planning

We divide procedures into three categories based on the bleeding risk as follows: “minimal,” “low,” and “high” risk. Table 4 provides an overview in which category common dentoalveolar procedures could fall. This overview excludes major maxillofacial surgery.

**Table 4:** Bleeding risk of dentoalveolar procedures (excluding major maxillofacial surgery)

	Minimal risk	Low risk	High risk
Anesthesia	Sedation techniques or general anesthesia 1	Local anesthesia (e.g., infiltration, intraligamental, and intrapulpal techniques) 13 Mandibular block injections 1 12 13	–
Oral hygiene	Cleaning (sub- or supragingival), pit and fissure sealing	Simple and deep scaling 12	–
Endodontic treatment	Instrumentation not beyond apical constriction	Pulpotomy 12 46 Instrumentation beyond apical constriction	Apical endodontic surgery
Periodontal treatment	–	Subgingival scaling and root planing	Periodontal surgery (curettage, (major) flap surgery 13 ) a
Tooth extractions	Singular single-rooted tooth extractions 46 49	Simple tooth extractions 35 49 99	>3 roots or complex procedures 13
Dental implants	–	Regular procedures 14 35 99 100	Mandibular procedures a
Oral surgery, other	–	(Superficial) abscess incision 35 99 Mucosal and mucocutaneous biopsies Biopsies of apical lesions 14 101	Surgical removal of teeth Deep tissue biopsies (e.g., salivary glands)
Prosthetics	Total prosthesis, prosthetic space maintainers	–	–
Crowns and bridgework	Uncomplicated procedures with minimal gingival manipulation	Procedures requiring subgingival manipulation/preparation or removal of dental cement	–
Restorative dentistry (“fillings”)	Uncomplicated procedures with minimal gingival manipulation	Procedures requiring gingival or subgingival manipulation	–
Orthodontics	Non-invasive orthodontic procedures	Procedures requiring gingival or subgingival manipulation	Orthodontic surgery
Posttraumatic procedures	–	Minimal invasive procedures (e.g., repositioning and splinting)	Any surgical procedure

a Local factors, such as concomitant periodontal disease, might increase the bleeding risk of the procedure.

Limiting the extent of the dentoalveolar procedure in patients with bleeding disorders could decrease the risk of (severe) bleeding and improve the possibilities to control any bleeding should it occur. 1 14 46 In this respect, treating mandibular procedures unilaterally should be considered, particularly if the platelet count is <50 × 10 ^9^ /L, as bilateral bleeding could threaten the airway.

When a considerable bleeding risk is anticipated, it is preferable to treat the patient early in the day to allow for observation. If feasible, it might also be considered to either postpone the procedure until the ITP is more stable or in remission or to avoid surgery altogether. It should be taken into account that postponing or avoiding surgery might be an inferior option, especially since bleedings seem to occur rarely. 1

In any patient with a bleeding tendency, we advocate putting effort into obtaining optimal local hemostasis. 9 Several principles have previously been recommended as mentioned below 1 14 46 :

- Delicate tissue handling and application of cautery, suturing. and wound packing (e.g., bite gauze).
- Avoiding major periodontal flap procedures when possible.
- Eliminating associated granulation tissue.
- Primary surgical closure, especially where flaps have been elevated.

The use of local hemostatic interventions (e.g., hemostatic sponges or fibrin sealants) and antifibrinolytics are recommended in ITP patients. The choice for the type of local hemostatic measures should be based on the specific circumstances and preferences of the treating oral professional, since no evidence is available in thrombocytopenic patients as to which treatment is most effective. 35 47 48 49 50

### Increasing the Platelet Count

As depicted in the flowchart ( Fig. 2 ), we recommend a minimal platelet count of more than 30 or 50 × 10 ^9^ /L for low- or high-risk procedures, respectively; no minimal platelet count is required for minimal risk procedures ( Table 4 ). However, a higher minimal platelet count could be appropriate in the presence of other bleeding risk factors such as a notable bleeding tendency (particularly presence of mucosal bleeds such as gum- or nosebleeds), 18 impaired platelet function, anticoagulants, and comorbidity. 9 15 16 17 18

In practice, we check the platelet count 2 weeks prior to the procedure to allow for an intervention, if necessary. If no intervention is required and the platelet count is generally stable, no additional check is necessary; otherwise, we check the platelet count on the day of the procedure.

Fig. 2 presents dentoalveolar procedure through flowchart.

The several therapies to boost a patient's platelet count include thrombopoietin receptor agonists (TPO-RAs) such as eltrombopag and romiplostim, IVIg, steroids, and platelet transfusion ( Fig. 2 ). Characteristics of the individual patient should always be considered when choosing the preoperative strategy. In general, TPO-RAs, either eltrombopag or romiplostim may be the first choice. Compared with IVIg, they are at least effective, 51 more convenient, less expensive, and they have relatively few side effects. 6 52 53 54 55 56 57 58 IVIg is an effective alternative option. Although IVIg is expensive and the patient-burden is high in terms of side effects and logistics (hospital visits), 59 60 61 62 63 64 65 66 67 68 69 its use is well known for this indication and the time to response is relatively short. 21 70 71 Steroids are cheap and often effective. However, their application should be reconsidered due to the side effects associated with even short courses of steroids including sleep disturbances, mood swings, glucose dysregulation and an increased risk of sepsis, venous thromboembolism, and fractures. 24 72 73 74 75 76 Platelet transfusion should be reserved for acute situations and very refractory patients; the effect is usually short lived and poses a risk of transfusion-related adverse events. 62 77 Although no evidence is available on this subject, based on the Dutch clinical practice, we generally suggest to start the procedure within 30 minutes after 2 units of platelets (minimal 2.5 × 10 ^11^ /L platelets per bag) have been administered. For practical reasons, a platelet count below the minimum, in this case, is regarded solely as a trigger for transfusion; the platelet count is not checked after transfusion to avoid time loss.

We recommend to not routinely check the platelet count postoperatively, unless a problem occurs. Postoperatively, the recently initiated medication can be discontinued. If the patient is already using ITP medication and the platelet levels are adequate, the treatment can be continued during the procedure. In patients using long-term low-dose steroids (<7.5 mg/day), it is not necessary to increase the dose for routine dental treatment, including minor surgical dentoalveolar procedures. 78

### Antifibrinolytic Therapy

With regard to antifibrinolytic therapy, we recommend the use of tranexamic acid orally (1–1.5 g two to three times daily) 35 49 which should be started several hours prior to the procedure and continued for 5 to 7 days. Antifibrinolytic therapy is contraindicated in case of severe kidney disease (estimated glomerular filtration rate <15 [mL/min/1.73m ^2^ ]), active thromboembolic disease, or a history of convulsions. 75 In addition, antifibrinolytic agents can be used topically as a mouthwash to be rinsed gently through the mouth or soaked in a sponge if the surgical site is accessible.

### Analgesics

Traditional nonsteroidal anti-inflammatory drugs (NSAIDs) inhibit the hemostatic function of platelets and should therefore be avoided in patients with thrombocytopenia. 79 80 81 Instead, the use of opioids could be considered or coxib's (selective NSAIDs, such as celecoxib and etoricoxib), since these agents primarily inhibit cyclooxygenase 2 (COX-2) rather than COX-1, and therefore affect platelet function less.

### Infection, Wound Healing, and Osteonecrosis

ITP patients potentially have an impaired wound healing and a higher risk of infection. The dental professional and/or treating physician should be aware of this possible increased risk, particularly in patients using corticosteroids or other immunosuppressive medication. There is insufficient evidence of a substantially increased infection risk to recommend the administration of prophylactic antibiotics routinely.

Vigilance is needed for osteonecrosis risk of the jaw in selected patients that had (previous) exposure to bisphosphonates. Preferably, necessary dentoalveolar procedures are performed before the start of the bisphosphonates. 82

### Discussion of the Institutional Guideline

The strength of this institutional guideline is that it is the only available guidance for clinical practice. Uniform practice will also enable future research regarding the safety and management of dentoalveolar procedures in ITP. A weakness of the guideline is that no formalized method was used.

As previously mentioned, no specific guidelines are available for managing dentoalveolar procedures in ITP patients. The 2003 ITP guidelines only recommend minimal platelet counts for some general procedures: 10 × 10 ^9^ /L for routine noninvasive dentistry, >30 × 10 ^9^ /L for tooth extractions, and regional mandibular blocks and >50 × 10 ^9^ /L for minor oral surgery. 21 These are in line with our recommendations in Table 4 and Fig. 2 . These 2003 ITP guidelines do not specifically mention methods to increase the platelet count prior to dentoalveolar procedures, other than that IVIg is an appropriate treatment for this indication. The 2019 ITP guidelines do not mention dentoalveolar procedures at all nor recommend platelet counts or treatments to perform other forms of surgery. 6 Lastly, we checked guidelines for oral and dental management in patients with myelodysplastic syndrome and acute myeloid leukemia. 83 These advise a platelet count of >50 × 10 ^9^ /L for dental care; there are no specifications regarding the type of procedure. The treatment options focus on platelet transfusion, because no other obvious options are available and there is no reason to expect a shortened half-life of the platelets in this patient category.

Future research, ideally, should focus on the development of formalized guidelines, for example, based on a Delphi study for which this guideline may serve as a basis or the Grading of Recommendations Assessment, Development and Evaluation (GRADE) approach when more research becomes available. 84 85 86 87 88

## Conclusion

This systematic review showed a serious lack of available evidence on the management of dentoalveolar procedures in ITP patients, especially for children. Clinically relevant bleeding and infection-related complications after dentoalveolar procedures in ITP patients seemed rare. Prospective studies are needed to further evaluate the safety of dentoalveolar procedures in ITP patients with low platelet counts, as well as the need for and efficacy of therapeutic interventions, to prevent bleeding and/or infections. Awaiting additional evidence to guide the development of evidence-based protocols, we have provided our institutional guideline to guide dental specialists and hematologists in the daily practice of managing dentoalveolar procedures in ITP patients.

## Notes

**What Is Known on This Topic?**

- Dentoalveolar procedures in immune thrombocytopenia (ITP) pose a risk of bleeding, due to thrombocytopenia, and infection, due to immunosuppressive treatments.
- There are currently no guidelines available to support hematologists and dental professionals in the management of dentoalveolar procedures in ITP patients.

**What Does This Paper Add?**

- This paper provides an overview of the available evidence regarding the risk of bleeding and infection in ITP, as well as the effectiveness of management strategies.
- To guide health care providers in the management of dentoalveolar procedures in ITP patients despite the limited available evidence, we provide our institutional guidelines.
