# Supplementary material for: Dentoalveolar Procedures in Immune Thrombocytopenia; Systematic Review and an Institutional Guideline
Source: TH Open. 2021 Sep 9;5(4):e489–502. doi: 10.1055/a-1641-7770 (PMC8595053; doi:10.1055/a-1641-7770)
Supplement: Supplementary file 1 — Supplementary Material [file 10-1055-a-1641-7770-s210030.pdf]

Supporting information

Supplemental tables

Supplemental table 1. Explanation of the dentoalveolar procedures and terminology.

Terminology	Short description
**Amalgam**	An alloy of mercury and silver, used as dental restorative and filling material, nowadays mainly replaced by composite (see below).
**Alveolus**	Tooth-bearing bony wall of the jaw.
**Apical treatments versus not beyond the apical constriction**	The apical constriction is the narrowest part of the apex and is located at the bottom part of the root of a tooth; apical treatments are treatments beyond the apical constriction.
**Composite reconstruction**	Restauration of a broken tooth using a composite filling material, that consists of a blend of tooth-colored plastic and powdered glass.
**Crown**	A (usually metal or ceramic) restorative cap placed over a damaged tooth.
**Debridement**	A procedure to remove plaque and calculus that have accumulated on the teeth.
**Dental implantation**	Tooth-root replacement by an artificial root (mostly consisting of titanium) that is placed into the jaw.
**Endodontic treatment**	Treatment involving the inside of the tooth, i.e. the dental pulp tissues present in pulp chamber and root canal.
**Fibrin sealants**	Agents comprised of human fibrinogen, human thrombin, and sometimes other added components, that mimic the final steps of the coagulation cascade to form a fibrin clot.
**Hemostatic sponge**	A resorbable spongiform material applied in a wound to stop a bleeding.
**Mandibular block**	Local anesthesia of the inferior alveolar nerve at the mandibular foramen.
**Mandibular fenestration (of simple bone cyst)**	A perforation in the mandibular bony cortex to reach a lesion in the mandibular marrow.
**Minimally traumatizing techniques**	Surgical techniques that aim to keep the surgical area (wound) as small as possible
**Pit and fissure sealing**	The closure of pits and fissures in the teeth with a thin plastic coating to prevent accumulation of food and bacteria and thus

	preventing caries.
**Primary closure**	The complete closure of a surgical wound using sutures, as opposed to healing by secondary intention.
**Pulpotomy**	A procedure in which the pulp tissue is removed out of the crown, i.e the visible part of the tooth, while leaving the pulp in the root canal intact; mainly done in children.
**Rootplaning**	Smoothing the exposed outer surfaces of the roots of the teeth, usually done after the root scaling (see below).
**Root scaling**	Removing dental plaque and calculus from the root of a tooth, either supra-gingival or sub-gingival.
**Splinting (of traumatically displaced alveolus and teeth)**	The application of a splint technique to fixate a mobile tooth after a trauma.

Supplemental table 2. Quality of descriptions

Quality of descriptions	Guzeldemir	Ingholo	Sangwan	Abdelwahab	Finucane	Vaisman	Tarantino	Tay	Oda	Harms	McKelvy	Martin	Lee	Bartholomew	Lario-nova	Suwannuraks	Rakocz	Baudo
(Group of) patient(s)	●●●	●●○	●●●	●○○	●●○	●●○	●○○	●●●	●●○	●●○	●●●	●●○	●●●	●●○	●●●	●○○	●○○	●○○
Indication for procedure	●●●	●●○	●●●	●○○	●●●	●●●	●○○	●●○	●●●	●●●	●●○	●●○	●●○	●○○	●●○	●○○	●○○	●○○
Procedure	●●●○	●●●●	●●○○	●○○○	●●●●	●●○○	●○○○	●●●●	●●●○	●●●●	●●●○	●●●●	●●○○	●○○○	●●●●	●○○○	●○○○	●○○○
Bleeding prevention measurements	●○○	●●●	●●○	●●○	●●○	●○○	●○○	●●○	●●○	●○○	●○○	●●○	●○○	●●○	●●○	●●●	●●●	●●●
Control group	NA	NA	NA	NA	NA	NA	●●○	NA	NA	NA	NA	NA	NA	NA	NA	NA	NA	NA
Follow-up	●●●	●○○	●●●	●○○	●●●	●●●	●○○	●●○	●●●	●○○	●●●	●●○	●●●	●●○	●○○	●●○	●○○	●○○
Outcome	●○○	●○○	●○○	●○○	●○○	●●○	●○○	●●●	●●○	●●○	●●○	●●●	●●●	●○○	●●●	●●●	●●○	●●○
**Total**	●●●	●●○	●●●	●○○	●●●	●●○	●○○	●●●	●●●	●●○	●●●	●●●	●●●	●●○	●●●	●●○	●●○	●●○

●●● = good (5 points), ●●○ = medium (3 points), ●○○ = insufficient or not reported (1 point)
●●●● = good (5 points), ●●●○ = adequate (4 points), ●●○○ = medium (2 points), ●○○○ = insufficient or not reported (1 point)
See for explanation of scores supplemental table 3.
NA = not applicable

Supplemental table 3. Risk of bias

	Abdelwahab	Tarantino	Baudo	Bartholomew	Rakocz	Suwannuraks
**Bias: confounding?**	1/5	2/5	1/5	1/5	1/5	1/5
**Could patients switch intervention groups?**	NA[a]	0/5	NA[a]	NA[a]	5/5	NA[a]
**Were intervention discontinuations or switches likely related to outcome?**	0/5	0/5	5/5	0/5	5/5	5/5
**Was statistically corrected for confounders?**	0/5	0/5	0/5	0/5	0/5	0/5
**Were potential confounders correctly measured?**	0/5	0/5	0/5	0/5	0/5	0/5
**Bias: good participant selection?**	5/5	1/5	5/5	5/5	5/5	5/5
**Was the intervention successfully implemented for most participants?**	0/5	5/5	5/5	5/5	5/5	5/5
**Were important co-interventions balanced across intervention groups?**	NA[a]	0/5	NA[a]	NA[a]	NA[a]	NA[a]
**Were outcome data available for (nearly) all patients?**	0/5	5/5	5/5	5/5	5/5	5/5
**Were participants excluded due to missing data on intervention status?**	0/5	0/5	5/5	5/5	5/5	5/5
**Were participants excluded due to missing data on other variables needed for analysis?**	0/5	0/5	5/5	5/5	5/5	5/5
**Are proportions and reasons for missing data similar across interventions?**	NA[a]	0/5	NA[a]	NA[a]	NA[a]	NA[a]
**Were outcome assessors aware of intervention?**	0/5	0/5	0/5	0/5	0/5	0/5
**Were the measures of outcome assessment comparable across intervention groups?**	NA[a]	0/5	NA[a]	NA[a]	NA[a]	NA[a]
**Total points**	**6/50 (12%)**	**13/70 (19%)**	**31/100 (31%)**	**26/100 (26%)**	**36/105 (34%)**	**31/100 (31%)**
**Risk of bias**	**High**	**High**	**Medium**	**High**	**Medium**	**Medium**

a) Only one intervention group. <30% =High risk of bias, 30-49% = Medium risk of bias, 50-69% = Acceptable risk of bias, ≥70% = Low risk of bias.
See for explanation of points supplemental table 4.

NA: not applicable.

Supplemental table 4. Explanation of critical appraisal

Quality of description						
	**1 point**	**1 point**	**2 points**	**3 points**	**4 points**	**5 points**
**Detailed description of patient (group)**	Not reported	Missing: PC at intervention or ≥3	-	Missing 1 or 2 (not: PC at intervention)	-	PC at intervention; concomitant medication; age; disease duration; splenectomy status
**Detailed description of indication for procedure**	Not reported	Missing: ≥3	-	Missing: 1 or 2	-	History, oral examination, dental problem leading to intervention, role of ITP
**Detailed description of procedure**	Not reported	Procedure vaguely	Procedure clearly but anesthetics not	-	Procedure clearly but anesthetics vaguely	Procedure + anesthetics clearly described
**Detailed description of bleeding prevention measurements**	Not reported	Not the specific substance or route of administration or not reported	-	No dose and/or duration or <3 of possible measures not specifically reported	-	Substance, route of administration, dose and duration
**Detailed description of control group**	Not reported	Missing: PC at intervention or ≥3	-	Missing 1-3 (no PC at intervention)	-	PC at intervention; concomitant medication; age; disease duration; splenectomy status
**Follow-up duration**	Not reported	<5 days	-	5-10 days	-	>10 days
**Detailed description of outcome**	Not reported	Bleeding or intervention not reported or missing ≥3	-	Missing: days after procedure or intervention not clearly described	-	Bleeding/infection, need for intervention + specifics, days after procedure, relevant other information
Quality of design						
	**0 points**	**1 point**	**2 points**	**3 points**	**4 points**	**5 points**
**Bias: confounding?**	-	Other bleeding/infection risk factors not mentioned	Other bleeding risk factors only vaguely mentioned; infection not or vaguely	-	Other bleeding risk factors specifically mentioned; infection only vaguely	Other bleeding (e.g. use of anticoagulants) and/or infection risk factors specifically mentioned (e.g. concomitant use of immunosuppressive agents)
**Could patients switch intervention groups**	Not reported or yes	-	-	-	-	No
**Were intervention discontinuations or switches likely related to outcome**	Not reported or yes	-	-	-	-	No
**Was statistically corrected for confounders**	Not reported or no	-	-	-	-	Yes
**Were potential confounders correctly measured?**	Not reported or no	-	-	-	-	Yes
**Bias: good participant selection?**	Not reported	Unclear why which patient gets which intervention	-	-	-	Predefined in- and exclusion criteria

**Was the intervention successfully implemented for most participants**	Not reported	<80%	-	-	-	≥80%
**Were important co-interventions balanced across intervention groups?**	No or not reported	-	-	-	-	Yes
**Were outcome data available for (nearly) all patients?**	Not reported or ≥60% missing	Available for 60-80%		-	Available for ≥80%	Available for ≥90%
**Were participants excluded due to missing data on intervention status?**	Yes or not reported	-	-	-	-	No
**Were participants excluded due to missing data on other variables needed for analysis?**	Yes or not reported	-	-	-	-	No
**Are proportions and reasons for missing data similar across interventions?**	No or not reported		-	-	-	Yes
**Were outcome assessors aware of intervention?**	Yes or not reported		-	-	-	No
**Total**	Percentage of total points: >80% = Good ; 70-79% = Acceptable ; 60-69% = Medium ; <60% = Insufficient					

ITP: immune thrombocytopenia, NA: not applicable, PC: platelet count

Supplemental table 5. Hemostatic strategies and anesthetic approach per platelet count category: adults

	Platelet count at time of procedure							Total per hemostatic therapy / anesthetic approach (row total)	Number of clinically bleedings, N (% of row total)
	Categories					*Reported otherwise*	*Not reported*		
	**<20** *10^9/L	**20-50** *10^9/L	**50-100** *10^9/L	**>150** *10^9/L	**10-70** [a] *10^9/L	**80** [b] *10^9/L (9-412)[a]			
**All procedures, N (% of row total (N=118))**	**13 (22)**	**2** [c,d] **(3)**	**4 (7)**	**1 (2)**	**6 (10)**	**32 (53)**	**2 (3)**	**60**	**2 (3)**
**Hemostatic therapy, N (% of column total)**									
**TXA (systemic + local)**									
Combined with surgical measures							1 (13)	1 (2)	0 (0)
**TXA (systemic)**									
Combined with surgical measures			1 (25)					1 (2)	0 (0)
**TXA (local)**									
Combined with surgical measures			1 (25)					1 (2)	0 (0)
Surgical measures NR			1 (25)					1 (2)	0 (0)
**Aminocaproic acid (systemic)**									
Surgical measures NR			1 (25)					1 (2)	0 (0)
**Only surgical measures**	11 (85)				6 (100)			17 (28)	0 (0)
**Not reported**	2 (15)	2 (100)		1 (100)		32 (100)	1 (88)	38 (63)	2[c,d] (5)
**Anesthetic approach, N (% of column total)**									
**No anesthesia**	1 (8)							1 (2)	0 (0)
**Local infiltration**	12 (92)		1 (25)				1 (50)	14 (23)	0 (0)
**General**				1 (100)				1 (2)	0 (0)
**Mandibular block**			1 (25)					1 (2)	0 (0)
**Not reported**		2 (100)	2 (50)		6 (100)	32 (100)	1 (38)	43 (72)	2[c,d] (5)

a) Range. b) Median. c) Clinically relevant non-major bleeding occurred. d) Major bleeding occurred.

NR: not reported, TXA: tranexamic acid.

Supplemental table 6. Hemostatic strategies and anesthetic approach per platelet count category: children

	Platelet count at time of procedure *Categories* <20 $*10^9/L$	20-50 $*10^9/L$	50-100 $*10^9/L$	100-150 $*10^9/L$	>150 $*10^9/L$	*Not reported*	Total per hemostatic therapy / anesthetic approach (row total)	Number of clinically bleedings, N (% of row total)
**All procedures, N (% of row total (N=118))**	**1 (8)**	**2 (15)**	**1 (8)**	**2 (15)**	**1 (8)**	**6 (46)**	**13**	**0 (0)**
**Hemostatic therapy, N (% of column total)**								
**TXA (systemic + local)**								
Combined with surgical measures	1 (100)						1 (8)	0 (0)
**Not reported**		2 (100)	1 (100)	2 (100)	1 (100)	6 (100)	12 (92)	0 (0)
**Anesthetic approach, N (% of column total)**								
**No anesthesia**		1 (50)				1 (17)	2 (15)	0 (0)
**Local infiltration**				1 (50)	1 (100)	3 (50)	5 (38)	0 (0)
**General**	1 (100)	1 (50)	1 (100)				3 (23)	0 (0)
**Not reported**				1 (50)		2 (33)	3 (23)	0 (0)

a) Range. b) Median. c) Clinically relevant non-major bleeding occurred. d) Major bleeding occurred.

NR: not reported, TXA: tranexamic acid.

Supplemental table 7. Characteristics per procedure.

Paper	Num-ber of cases	Age at proce-dure (yrs)	Procedure description	PC at proce-dure $*10^9/L$	Bleeding preventive measures		Major blee-dings
					To raise PC pre-op	Other hemostatic measures	
**Adults**							
**Baudo**[48]	4	NR	Dental extractions *N=6*	10-70	None	Peri-op: fibrin sealants<br>Post-op: none	No
**Bartholomew**[49]	1	33	Dental extractions	81	Prednisone (dose NR)	Peri-op: NR<br>After: aminocaproic acid oral (4 g/day for 4 days)	No
**Harms**[54]	1	43	Dental extraction, minimally invasive	12	NR	NR	No
**Inchingolo**[55]	1	20	Dental extraction (impacted)	60	None	Peri-op: TXA IV (4 vials before procedure, 4 vials during procedure; 1 vial was 500ml 4.8%) + surgicel<br>Post-op: TXA IV (4 vials)	No
**Larionova**[56]	1	26	Re-endodontic treatment + incision of subperiosteal abscess using laser	2	Prednisone IV (dose NR)	NR	No
**Lee**[34]	1	55	Dental extraction (left mandibular first molar)	35	None	NR	Yes
		+30 [d]	Dental implantation (left mandibular first molar)	22[a]	None	NR	Yes *(life threatening)*
		+120 [d]	Prosthetic implant restoration	NR	NR	NR	No*
**Martin**[57]	1	70	Dental extraction	55	Eltrombopag (50 mg/day)	Peri-op: hemostatic sponge<br>Post-op: TXA mouthwash	No

Commented [WvD1]: 5.R. Soltani, A. Haghighat, M. Fanaei, and G. Asghari, "Evaluation of the effect of green tea extract on the prevention of gingival bleeding after posterior mandibular teeth extraction: a randomized controlled trial," Evidence-based Complementary and Alternative Medicine, vol. 2014

Commented [WvD2]: The dentist was unfamiliar with ITP; dus waarschijnlijk niet

**McKelvy**[58]	1	42	Dental extractions (≤3)	250	NR	NR	No
**Rakocz**[50]	6	NR	Dental extractions, minimal trauma *N=11*	<15	None	Peri-op: fibrin sealants Post-op: none	No
**Sangwan**[60]	1	19	Debridement, scaling and rootplaning	72	Prednisone (dose NR)	Peri-op: NR Post-op: TXA mouthwash	No
**Suwannuraks**[51]	1	NR	Dental extractions, minimal trauma	NR	NR	Peri-op: fibrin sealants Post-op: TXA mouthwash + oral (10 mg/kg, 3x/day for 7 days), celluloid splint	No
**Tarantino**[31]	24	NR	A range of procedures, including cleaning, crowns, prosthetics, dental extraction, and endodontics *N=32*	80 (range 9-412)	Danzol + platelet transfusion[b] (doses NR) IVIg[b] Methylpredniso-lone[b] (doses NR)	NR	No
**Children**							
**Finucane**[52]	1	13	Repositioning and splinting of traumatically displaced alveolus and teeth	70	IVIg (1 g/kg for 2 days)	NR	No
		NR	Endodontic treatment of nonvital replaced teeth	NR	NR	NR	Loss to FU
**Guzeldemir**[53]	1	11	Cleaning (neglected oral health)	NR	NR	NR	No
		+14[d]	Scaling and rootplaning	144	NR	NR	No
		+63[d]	Scaling and rootplaning	180	NR	NR	Loss to FU
**Oda**[59]	1	12	Dental extraction + incisional biopsy**	NR	IVIg (400 mg/kg for 5 days) + platelet transfusion (dose NR)	NR	No
		+30[d]	Dental extraction + mandibular fenestration**	NR	Platelet transfusion (dose NR)	NR	No

		19	Dental extractions (≤3) + mandibular fenestration**	43	IVIg (400 mg/kg for 5 days) + platelet transfusion (dose NR)	NR	No
**Tay**[61]	1	13	Dental extraction + cleaning and scaling	4	None[$]	Peri-op: TXA sponges + collagen derivates<br>Post-op: TXA oral (500mg 3x/day for 5 days)	No
**Vaisman**[62]	2	5	Composite reconstruction + pulpotomy	30[$$]	NR	NR	No
		+ *NR*	Final rehabilitation (crowns, prosthetic space maintainers)	130	Prednisone (dose NR)	NR	No
		11	Pit and fissure sealing + composite restorations + orthodontics	NR	NR	NR	No
		7	Dental extractions (>3) + composite reconstruction + amalgam restoration + pulpotomy and crown	NR	NR	NR	No

*** Underlying hemorrhagic bone cyst*
$ Refractory of IVIg and prednisone, but this was available as rescue therapy
$$ Measured postoperatively because of minor bleeding
a. measured post-procedural in emergency room
b. n=1
c. requiring blood transfusion
d. number of days after 1st procedure
FU: follow-up, N: number, PC: platelet count, L: liter, pre-op: preoperatively, peri-op: perioperatively, post-op: postoperatively, NR: not reported, N/A: not applicable, IVIg: intravenous immunoglobulins, TXA: tranexamic acid, IV: intravenous

Search details

PUBMED

("Purpura, Thrombocytopenic, Idiopathic"[Mesh] OR "immune thrombocytopenia" OR "immune thrombopenia" OR "immune thrombocytopenic" OR "immune thrombopenic" OR "autoimmune thrombocytopenia" OR "autoimmune thrombopenia" OR "autoimmune thrombocytopenic" OR "autoimmune thrombopenic" OR "autoimmune thrombocytopenic purpura" OR "idiopathic thrombocytopenia" OR "idiopathic thrombopenia" OR "idiopathic thrombocytopenic" OR "idiopathic thrombopenic" OR Werlhof OR "immune thrombocytopenic purpura" OR "idiopathic thrombocytopenic purpura") AND (Dental OR dentist* OR dentition OR periodontal OR periodontic* OR prosthodontic OR prosthodontics OR orthodontic OR endodontic OR endodontics OR tooth OR teeth OR molar OR molars OR "oral surgery" OR "oral surgical procedures" OR "operative dentistry" OR dentoalveolar OR orthognathic OR "surgery, oral"[MeSH Terms] OR "oral surgical procedures"[MeSH Terms] OR "tooth extraction"[MeSH Terms] OR "Dentistry, Operative"[MeSH] OR "molar"[MeSH Terms] OR "Dentists"[Mesh] OR "Dentistry"[Mesh] OR "Dentition"[Mesh])

EMBASE

('idiopathic thrombocytopenic purpura'/exp OR 'immune thrombocytopenia' OR 'immune thrombopenia' OR 'immune thrombocytopenic' OR 'immune thrombopenic' OR 'autoimmune thrombocytopenia' OR 'autoimmune thrombopenia' OR 'autoimmune thrombocytopenic' OR 'autoimmune thrombopenic' OR 'idiopathic thrombocytopenia' OR 'idiopathic thrombopenia' OR 'idiopathic thrombocytopenic' OR 'idiopathic thrombopenic' OR Werlhof) AND (Dental OR dentist* OR dentition OR periodontal OR periodontic* OR prosthodontic OR prosthodontics OR orthodontic OR endodontic OR endodontics OR tooth OR teeth OR molar OR molars OR 'oral surgery' OR 'oral surgical procedures' OR 'operative dentistry' OR dentoalveolar OR orthognathic OR 'oral surgery'/exp OR 'tooth extraction'/exp OR 'dental procedure'/exp OR 'dentistry'/exp OR 'dentition'/exp)

CINAHL

("immune thrombocytopenia" OR "immune thrombopenia" OR "immune thrombocytopenic" OR "immune thrombopenic" OR "autoimmune thrombocytopenia" OR "autoimmune thrombopenia" OR "autoimmune thrombocytopenic" OR "autoimmune thrombopenic" OR "autoimmune thrombocytopenic purpura" OR "idiopathic thrombocytopenia" OR "idiopathic thrombopenia" OR "idiopathic thrombocytopenic" OR "idiopathic thrombopenic" OR Werlhof OR "immune thrombocytopenic purpura" OR "idiopathic thrombocytopenic purpura") AND (Dental OR dentist* OR dentition OR periodontal OR periodontic* OR prosthodontic OR prosthodontics OR orthodontic OR endodontic OR endodontics OR tooth OR teeth OR molar OR molars OR "oral surgery" OR "oral surgical procedures" OR "operative dentistry" OR dentoalveolar OR orthognathic OR (MH "Surgery, Oral+") OR (MH "Dentistry+") OR (MH "Dentistry, Operative+") OR (MH "Tooth Extraction") OR (MH "Molar+") OR (MH "Tooth+") OR (MH "Dentists+") OR (MH "Dentition+"))

Cochrane

(‘immune thrombocytopenia’ OR ‘immune thrombopenia’ OR ‘immune thrombocytopenic’ OR ‘immune thrombopenic’ OR ‘autoimmune thrombocytopenia’ OR ‘autoimmune thrombopenia’ OR ‘autoimmune thrombocytopenic’ OR ‘autoimmune thrombopenic’ OR ‘idiopathic thrombocytopenia’ OR ‘idiopathic thrombopenia’ OR ‘idiopathic thrombocytopenic’ OR ‘idiopathic thrombopenic’ OR ‘Werlhof’) AND (‘Dental’ OR ‘dentist*’ OR ‘dentition’ OR ‘periodontal’ OR ‘periodontic*’ OR ‘prosthodontic’ OR ‘prosthodontics’ OR ‘orthodontic’ OR ‘endodontic’ OR ‘endodontics’ OR ‘tooth’ OR ‘teeth’ OR ‘molar’ OR ‘molars’ OR ‘oral surgery’ OR ‘oral surgical procedures’ OR ‘operative dentistry’ OR ‘dentoalveolar’ OR ‘orthognathic’)
